# Cascade of care among hepatitis B patients in Maastricht, the Netherlands, 1996 to 2018

**DOI:** 10.1016/j.jve.2022.100075

**Published:** 2022-06-20

**Authors:** Eva van Oorschot, Özgür M. Koc, Astrid ML. Oude Lashof, Inge HM. van Loo, Robin Ackens, Dirk Posthouwer, Ger H. Koek

**Affiliations:** a Faculty of Health, Medicine and Life Sciences, Maastricht University, Maastricht, the Netherlands; b Department of Internal Medicine, Division of Gastroenterology and Hepatology, Maastricht University Medical Centre, Maastricht, the Netherlands; c Department of Medical Microbiology, School of Nutrition and Translational Research in Metabolism (NUTRIM), Maastricht University Medical Centre, Maastricht, the Netherlands; d Department of Gastroenterology and Hepatology, Ziekenhuis Oost-Limburg, Genk, Belgium; e Faculty of Medicine and Life Sciences, Hasselt University, Hasselt, Belgium; f Department of Internal Medicine, Division of Infectious Diseases, Maastricht University Medical Centre, the Netherlands; g Care and Public Health Research Institute (CAPHRI), Maastricht University, the Netherlands; h Department of Integrated Care, Maastricht University Medical Centre, the Netherlands; i School of Nutrition and Translational Research in Metabolism (Nutrim), Maastricht University, the Netherlands; j Department of Visceral and Transplantation Surgery, Klinikum, RWTH, Aachen, Germany; k Department of Internal Medicine Division of Gastroenterology and Hepatology, Maastricht University, the Netherlands

**Keywords:** Hepatitis B, Cascade of care, Linkage to care, Loss to follow-up, Ethnicity, The Netherlands

## Abstract

**Background & aims:**

There are approximately 49,000 people (0.34%) in the Netherlands with a chronic hepatitis B virus (HBV) infection. It is unclear how many are linked to care and under follow-up in hepatitis outpatient clinics. This study determined the cascade of care and identified predictors for not being linked to care and loss to follow-up in Maastricht, the Netherlands.

**Methods:**

All hepatitis B surface antigen (HBsAg)-positive patients between December 1, 1996 and September 30, 2018 were retrospectively identified.

**Results:**

In total, 644 HBsAg-positive patients were identified; of whom 75 had acute HBV infection, 471 chronic HBV infection and 98 unknown. Out of 569 individuals with a chronic/unknown HBV status, 134/569 (23.6%) were not linked to care and 58.7% (195/332 after excluding those who died or achieved HBsAg-seroclearance) were loss to follow-up (LTFU). A predictor for not being linked to care was Caucasian ethnicity (odds ratio (OR) = 2.76 (95% Confidence Interval (CI) = 1.21–6.29); *p* = .015). Predictors for LTFU were older age (OR = 0.97 (CI = 0.94–0.99); *p* = .008), HBV DNA >20,000 IU/mL (OR = 0.44 (CI = 0.21 - 0.93); *p* = .033) and Asian ethnicity (OR = 0.46, (CI = 0.21–1.00); *p* = .050). Rates of not being linked to care and LTFU decreased over time from 12.7% in 1996 to 4.4% in 2018 and from 79.2% in 1996 to 37.2% in 2018, respectively.

**Conclusions:**

A considerable amount of HBsAg-positive individuals were not linked to care or LTFU. This study indicates that ethnicity plays a role in linkage to care and follow-up. Further research is needed to elaborate on those results.

## Abbreviations

ALT: alanine aminotransferase
Anti-HBc: hepatitis B core antibodies
CEUS: contrast-enhanced ultrasound
CI: confidence interval
CT: computed tomography
HBeAg: Hepatitis B e Antigen
HBsAg: hepatitis B surface antigen
HBV: hepatitis B virus
HCC: hepatocellular carcinoma
HCV: hepatitis C virus
HDV: hepatitis D virus
IDU: intravenous drug use
IQR: interquartile range
IU: international units
LTFU: Loss to follow-up
MRI: magnetic resonance imaging
MSM: men who have sex with men
MUMC: Maastricht University Medical Centre
NAFLD: Non-Alcoholic Fatty Liver Disease
OR: odds ratio
SD: standard deviation
SE: standard error
SPSS: Statistical Package for the Social Sciences
ULN: upper limit of normal
WHO: World Health Organization

## 1 Introduction

Infection with hepatitis B virus (HBV) can lead to necroinflammation of the liver. Clearance of the virus is achieved spontaneously in 90–95% of patients infected at adulthood, but patients who are infected during childhood are much more likely to develop chronic disease.^1^ Worldwide, there are an estimated 257 million people chronically infected with HBV.^2^ The global mortality of viral HBV is estimated to be 887,000 in 2015. This number is mainly the consequence of complications such as cirrhosis and hepatocellular carcinoma (HCC).^3^^,^^4^ In the development of chronic HBV infection, infected individuals frequently stay asymptomatic for decades before presenting with cirrhosis or HCC.^5^^,^^6^

Patients with chronic HBV infection should be linked to care for the evaluation of antiviral therapy. Treatment options for HBV have tremendously improved over the past 20 years. New therapies induce HBV DNA suppression in over 90% of the treated individuals. This will probably lead to fewer cases of cirrhosis, HCC and overall liver-related mortality in the near future.^7^^,^^8^ Furthermore, the newer oral antiviral therapies are well tolerated and cost-effective. Patients, not eligible for treatment according to the international HBV management guidelines, should be carefully monitored for progression to chronic active hepatitis, cirrhosis and HCC.^9^^,^^10^

By estimate, there are 49,000 people in the Netherlands chronically infected with HBV.^11^ Amongst this population, it is not clear how many have been linked to care and lost to follow-up.^6^^,^^12^, ^13^, ^14^ Therefore, this study aimed to determine the cascade of care in chronic HBV patients and to identify predictors for not being linked to care and predictors for loss to follow-up within a low HBV endemic region in Western Europe to ensure retainment into care and prevent long term-complicaties such as cirrhosis and HCC.

## 2 Methods

### 2.1 Study design and study population

We conducted a single centre, retrospective study to identify all patients with a positive hepatitis B surface antigen (HBsAg) test result between December 1, 1996 and September 30, 2018 within the region of Maastricht, the Netherlands. The results were compiled from electronic laboratory records. The Maastricht University Medical Centre (MUMC+) is the only hospital in Maastricht. Chronic HBV infection was defined as more than 6 months HBsAg positivity, measured by two or more positive HbsAg results, at least 6 months apart.^9^ In line with the definitions of the European Centre for Disease Prevention and Control (ECDC), patients with the appropriate symptoms/signs and laboratory confirmation were identified as definite acute hepatitis B cases.^15^ Laboratory confirmation included 1) a positive test for HBsAg and HBV core antibodies (anti-HBc) IgM, 2) detection of HBsAg and previous negative HBV markers or, 3) detection of HBV DNA and previous negative HBV markers.^15^ Unknown cases were those without laboratory confirmation and a HBsAg-positivity < 6 months.^16^

### 2.2 Outcome measures

The aim of this study was to gain an overview in the cascade of care for chronic HBV patients in the Maastricht region. Positive HBsAg patients were classified as acute, chronic and unknown, as previously outlined. We assessed the percentage of patients who had the recommended laboratory testing within 6 months: alanine aminotransferase (ALT), HBV DNA and hepatitis B e Antigen (HBeAg)).^17^ Acute cases were excluded in the subsequent analyses with regard to predictors for not being linked to care and loss to follow-up. Being linked or not linked to care was defined as chronic/unknown cases with or without an infectious disease specialist/hepatologist evaluation, respectively. Among those patients linked to care, eligibility for treatment was assessed according to the EASL guidelines.^9^ HBV DNA suppression was defined as having an unquantifiable HBV DNA based on sensitive PCR assay.^18^^,^^19^ Patients who were not candidates for antiviral therapy were considered to be loss to follow-up when assessment within 1 year was lacking.^20^ We also studied the trend over time from 1996 to 2018 in the proportion of individuals not linked to care and loss to follow-up.

Information regarding demographic, viral and disease outcome factors were collected using a standard form. Demographic factors included age at diagnosis, year of diagnosis, sex, ethnicity, and approximated duration of infection (calculated from diagnosis until last visit at the outpatient clinic). Alcohol abuse was defined as >14 units/week for men and >7 units/week for women and we included data regarding smoking, as well as the presence of comorbidities.^21^ Data on illicit drug use, intravenous drug use (IDU) and imprisonment were collected on lifetime prevalence and within the last six months of follow-up (<6 months). Viral factors comprised of HBV DNA level (categorized in <2,000, 2000–20,000, >20,000 IU/mL), HBeAg status, alanine aminotransferase (ALT) > upper limit of normal (ULN) level, co-infections (hepatitis C virus (HCV), HIV or hepatitis D virus (HDV)) and fibrosis, measured by transient elastrography in kPa at baseline.^22^, ^23^, ^24^

### 2.3 Statistical analyses

Categorical data were analysed with the Chi-squared test or Fisher's exact test. In case the observed value was zero, a Laplace correction was applied, i.e. adding one in all cells yielding a more honest test procedure.^25^ Differences in two continuous variables were assessed by the independent *t*-test. The Kolmogorov-Smirnov test and Levene's test were used to test normal distribution and homogeneity of variance, respectively. When violating the assumptions for parametric tests, the Mann-Whitney *U* test was used instead for comparing two continuous variables. Stepwise forward conditional multiple logistic regression analysis was performed to determine independent predictors for not being linked to care and loss to follow-up. Variables with a significant association (*p* <.10) in the univariate analyses were included in the multiple logistic regression model. Results were presented as either with frequencies (%), median ± interquartile range (IQR) or mean ± standard deviation (SD) as appropriate. Anonymous data collection and analyses were performed using Statistical Package for the Social Sciences (SPSS) (Release 23, Armonk, NY). The level of statistical significance was set at *p* < .05 on a two-sided test.

### 2.4 Ethics approval

Due to the observational character of the study, ethical approval was waived by the local ethics committee of Maastricht UMC+ (METC 2018–0334). Data lock was on 22-02-2019.

## 3 Results

### 3.1 Study population

**Fig. 1:**
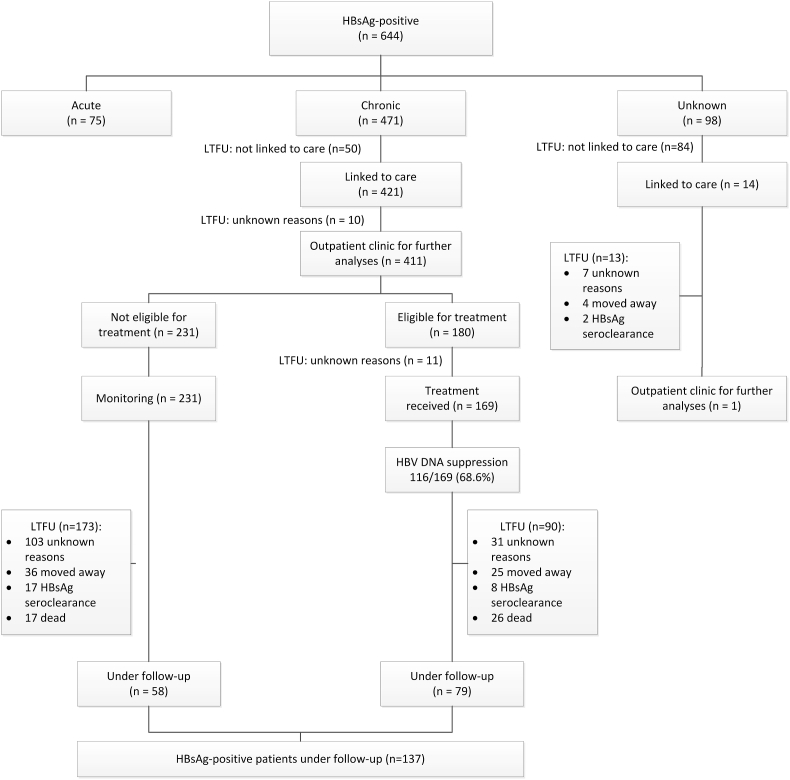
Cascade of care for HBsAg-positive patients within the region of Maastricht, The Netherlands (n=644) Abbreviations: HBsAg: Hepatitis B surface Antigen; LTFU: Loss to follow-up. Definitions: Chronic HBV infection: more than 6 months of HBsAg positivity; Acute HBV infection: patients with the appropriate symptoms/signs and laboratory confirmation: 1) a positive test for HBsAg and hepatitis B core antibodies (anti-HBc) IgM, 2) detection of HBsAg and previous negative markers or, 3) detection of HBV DNA and previous negative HBV markers; Unknown cases: those without laboratory confirmation and a HBsAg-positivity < 6 months. Linked to care: HBsAg-positive patients with an infectious disease specialist or hepatologist evaluation; Eligible for treatment: according to the EASL guidelines; loss to follow-up: no specialist evaluation >1 year with previous evaluation.

During the study period, a total of 644 people tested positive for HBsAg. Of those 644 patients, 75 (11.7%) were identified as acute hepatitis B, 471 (73.1%) had chronic HBV infection and 98 (15.2%) were unknown (Fig. 1). After excluding the 75 patients with acute hepatitis B from further analyses, 569 chronic/unknown HBV cases remained. The median age of the chronic/unknown HBV population (n=569) was 35 ± 20.0 years. They were predominantly male (59.1%) and Caucasian (54.2%). Table 1 illustrates the baseline characteristics of the study population. Thirteen percent (58 out of 445 with information on drug use) and 5.9% of the patients (26 out of 443 with information on intravenous drug use) ever used illicit drugs and intravenous drugs, respectively. Alcohol abuse was seen in 14.9% of the patients (66 out of 442 with information on alcohol use) and 3.7% of the patients (17 out of 461 with information on imprisonment) were ever imprisoned. In our chronic/unknown population, the overall mortality and liver-related mortality were 14.4% and 4.7% (75 out of 521 and 25 out of 515 patients) respectively in a mean follow-up duration of 6.44 ± 7.3 years.

**Table 1:** Baseline characteristics of patients with chronic or unknown hepatitis B virus infection categorized as linked or not linked to care (n = 569).

Characteristics	Total (n=569)	Linked to care (n=435)	Not linked to care (n=134)	p-value
***Demographic factors***				
**Age (years)**	35 ± 20.0	36 ± 20.0	33 ± 17.0	.383
**Male**	336/569 (59.1)	266/435 (61.1)	70/134 (52.2)	.067
**Ethnicity**				
• **Caucasian**	254/468 (54.2)	202/393 (51.4)	52/75 (69.3)	**.004**
• **Asian**	111/468 (23.7)	102/393 (26.0)	9/75 (12.0)	**.009**
• **African**	94/468 (20)	80/393 (20.4)	14/75 (18.7)	.738
• **Hispanic**	9/468 (1.9)	9/393 (2.3)	0/75 (0)	.205
**Illicit drug use ever**	58/445 (13.0)	52/398 (13.1)	6/47 (12.8)	.954
**IDU ever**	26/443 (5.9)	23/398 (5.8)	3/45 (6.7)	.505
**IDU < 6 months**	2/159 (1.3)	2/145 (1.4)	0/14 (0)	.813
**Imprisonment ever**	17/461 (3.7)	16/408 (3.9)	1/53 (1.9)	.398
**Alcohol abuse ever**	66/442 (14.9)	59/396 (13.6)	7/46 (15.2)	.954
**Alcohol abuse < 6 months**	5/137 (0.9)	5/137 (3.6)	N/A	N/A
**Smoking**	187/441 (42.4)	166/394 (42.1)	21/47 (44.7)	.738
**Homeless**	21/466 (4.5)	20/412 (4.9)	1/54 (1.9)	.276
**MSM**	33/454 (7.3)	33/371 (8.9)	0/83 (0)	**.005**
**Comorbidity**				
• **NAFLD**	91/404 (22.5)	35/376 (8.6)	6/28 (21.4)	.886
• **Diabetes mellitus**	39/460 (8.5)	35/409 (8.6)	4/51 (7.8)	.560
• **Hypertension**	118/466 (25.3)	101/412 (24.5)	17/54 (31.5)	.268
***Viral factors***				
**HBeAg status positive**	101/464 (21.8)	83/354 (23.4)	18/110 (16.4)	.116
**ALT level > ULN**	181/445 (40.7)	161/380 (42.4)	20/65 (30.8)	.079
**HBV DNA (IU/mL)**				
• **< 2000**	136/257 (52.9)	125/237 (52.7)	11/20 (55.0)	.846
• **2000**–**20,000**	34/257 (13.2)	32/237 (13.5)	2/20 (10.0)	.657
• **> 20,000**	87/257 (33.9)	80/237 (33.8)	7/20 (35.0)	.910
**Fibrosis (kPa)**	6 ± 4.5	8 ± 4.5	N/A	N/A
**Co-infections**				
• **HCV**	35/402 (8.7)	32/351 (9.1)	3/53 (5.9)	.325
• **HIV**	60/342 (17.5)	58/289 (20.1)	2/51 (3.8)	**.004**
• **HDV**	8/147 (5.4)	8/147 (5.4)	N/A	N/A

Abbreviations: IDU: Intravenous Drug Use; IU: international units; MSM: Men who have Sex with Men; NAFLD: Non-Alcoholic Fatty Liver Disease; HBeAg: Hepatitis B e Antigen; ALT: alanine aminotransferase; ULN: Upper Limit of Normal; HBV: Hepatitis B Virus; HCV: Hepatitis C virus; HDV: Hepatitis D Virus.

All values are given as frequencies n (%) or median ± IQR.

Alcohol abuse was defined as > 14 units/week for men and >7 units/week for women.^21^

Compared to Asians (n = 111), Caucasian (n = 254) patients were significantly older (41 ± 21.0 vs 36 ± 20.0, *p =* .026) and more patients were male (172/254 (67.7%) vs 54/111 (48.6%), *p* = .001). In the Caucasian group, significantly more patients were MSM (25/206 (12.1%) vs 2/100 (2.0%), *p* = .003), illicit drug users (39/218 (17.9%) vs 4/99 (4.0%), *p* = .001), IDU (22/217 (10.1%) vs 1/99 (1.0%), *p* = .002), homeless (14/228 (6.1%) vs 1/101 (1.0%), *p* = .044) and co-infected with HIV (32/148 (14.6%) vs 5/71 (7.0%), *p* = .007) compared to the Asian group.

### 3.2 Cascade of care

Out of 644 HBsAg-positive individuals, 497 (77.2%) had ALT testing, 271 (42.1%) had testing for HBV DNA and 515 (80.0%) had HBeAg testing within 6 months of HBV diagnosis. Out of 569 patients with chronic/unknown HBV infection, 134 (23.6%) were not linked to care. Out of 134 (50 chronic and 84 unknown) patients without linkage to care, 46 (34.3%), 27 (20.1%) and 13 (9.7%) of the HBsAg tests were requested by general practitioners, gynaecologists/obstetricians and other clinics, respectively. Of our linked patients - after excluding those who died or achieved HBsAg loss during follow-up - 137/332 (41.3%) remained in follow-up. The cascade of care for the total HBsAg positive study population is illustrated in Fig. 1.

**Fig. 2:**
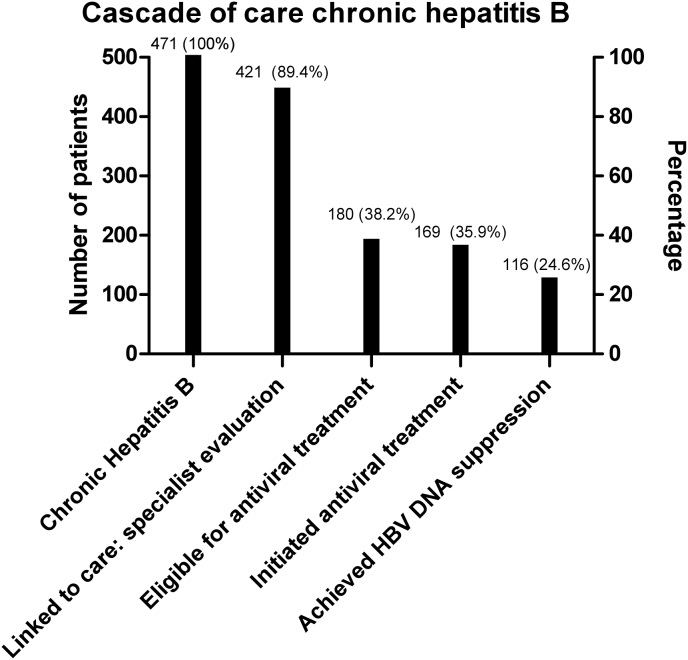
Cascade of care in chronic hepatitis B patients presented as columns (n=471) Abbreviations: HBV: Hepatitis B virus. Definitions: Chronic HBV infection: more than 6 months of HBsAg positivity; Linked to care: HBsAg-positive patients with an infectious disease specialist or hepatologist evaluation; Eligible for treatment: according to the EASL guidelines; HBV suppression: HBV DNA of <60–80 IU/ml.

When limited to the 471 chronic HBV patients, 50/471 (10.6%) patients were not linked to care. Out of 421/471 (89.4%) patients that were linked to care, 411/471 (87.3%) patients went to our outpatient clinic for further analyses of ALT, HBV DNA and HBeAg status, 180/471 (38.2%) seemed eligible for treatment and 169/471 (35.9%) received treatment. Out of 169 patients who received treatment, 56 (33.1%) were loss to follow-up, 25/56 (44.6%) moved away and in 31/56 patients (55.4%) reasons for loss to follow-up were unknown. In the case of 169 eligible patients who received treatment, 116 (68.6%) achieved HBV DNA suppression; 21/116 (18.1%) did so after initiating an interferon-based regimen and 95/116 (81.9%) were treated with nucleos(t)ide analogues. Currently, a total of 79/169 (46.7%) patients are under treatment and under active follow-up. Overall, 231/471 patients (49.0%) were not candidates for antiviral treatment and were monitored with periodical assessments. At present, 58/231 (25.2%) patients are currently being monitored. Fig. 2 illustrates cascade of care in patients with chronic HBV infection.

### 3.3 Predictors for not being linked to care

In our study population of chronic/unknown (n=569), a comparison was made between patients with and without linkage to care. Univariate analyses indicated that Caucasian ethnicity (202/393 (51.4%) linked to care vs 52/75 (69.3%) not linked to care, *p* = .004) was associated with not being linked to care. In contrast, Asian ethnicity (102/393 (26.0%) vs 9/75 (12.0%), *p* =.009), MSM (33/371 (8.9%) vs 0/83 (0%) *p* = .005) and HIV co-infection (58/289 (20.1%) vs 2/51 (3.8%), *p* =.004) were correlated with being linked to care (Table 1). Forward stepwise multiple logistic regression analysis indicated that Caucasian ethnicity was the only independent predictor for not being linked to care (odds ratio (OR) = 2.76, 95% confidence interval (CI) 1.21–6.29, *p* = .015) (Table 2).

**Table 2:** Stepwise forward analyses in patients with chronic/unknown hepatitis B virus infection for not being linked to care as outcome variable.

Factors significantly associated with not being linked to care on univariate analysis				P-value		
**Caucasian ethnicity**				.004		
**Asian ethnicity**				.009		
**HIV co-infection**				.004		
**MSM**				.005		
**Stepwise forward analysis**		**B**	**SE**	***p***	**OR**	**95% CI**
**Step 1**	**Constant**	.-2.85	.34	.000		
	**Caucasian**	.88	.42	.035	2.409	1.06–5.46
**Step 2**	**Constant**	−2.80	.34	.000		
	**Caucasian**	1.02	.42	**.015**	2.764	1.21–6.29
	**MSM**	−19.25	7151.90	.998	.000	.000-

Abbreviations: MSM: Men who have Sex with Men; CI: confidence interval; IU: international units; SE: standard error; OR: Odds Ratio.

Factors excluded in the forward stepwise analyses are:, HIV co-infection, Asian ethnicity..

### 3.4 Predictors for loss to follow-up

When exploring our chronic hepatitis B patients (n= 332, after excluding those who died or achieved HBsAg seroclearance), imprisonment (1/134 (0.7%) vs. 9/185 (4.9%), *p* = .033) was positively associated with loss to follow-up in univariate analyses. However, Asian ethnicity (46/129 (35.7%) vs. 40/174 (23.0%), *p* = .016), hypertension as comorbidity (40/137 (29.2%) vs. 30/185 (16.1%), *p* = .005), HBV DNA level >20,000 IU/mL (38/97 (39.2%) vs. 25/106 (23.6%), *p* = .016) and a positive HBeAg status (34/119 (28.6%) vs 29/157 (18.5%), *p* = .048) were negatively associated with loss to follow-up (Table 3). In a forward stepwise multiple logistic regression, age (OR = 0.97, 95% CI 0.94–0.99, *p* = .008), HBV DNA level >20,000 IU/mL (OR = 0.44, 95% CI .21 - .93, *p* = .033) and Asian ethnicity (OR = 0.46, 95% CI 0.21–1.00, *p* = .050) were identified as independent negative predictors for loss to follow-up (Table 4).

**Table 3:** Baseline characteristics of patients with chronic/unknown hepatitis B virus infection who are linked to care; by patients in follow-up vs. patients loss to follow-up (n = 332).

Characteristics	Total (n=332)	In follow-up (n=137)	Loss to follow up (n=195)	p
***Demographic factors***				
**Age (years)**	34 ± 19.0	39 ± 21.0	32 ± 15.0	**.002**
**Male**	191/332 (57.5)	78/137 (56.9)	113/195 (57.9)	.854
**Ethnicity**				
• **Caucasian**	140/303 (46.2)	52/129 (40.3)	88/174 (50.6)	.085
• **Asian**	86/303 (28.4)	46/129 (35.7)	40/174 (23.0)	**.016**
• **African**	69/303 (22.8)	26/129 (20.2)	43/174 (24.7)	.350
• Hispanic	8/303 (2.6)	5/129 (3.9)	3/174 (1.7)	.213
**Illicit drug use ever**	30/311 (9.6)	10/134 (7.5)	20/177 (11.3)	.256
**IDU (ever)**	8/311 (2.6)	2/134 (1.5)	6/177 (3.4)	.251
**IDU (< 6 months)**	2/144 (1.4)	2/134 (1.5)	N/A	N/A
**Imprisonment ever**	10/319 (3.1)	1/134 (0.7)	9/185 (4.9)	**.033**
**Alcohol abuse ever**	34/310 (11.0)	14/135 (10.2)	20/175 (11.4)	.768
**Alcohol abuse < 6 months**	5/135 (3.7)	5/135 (3.7)	N/A	N/A
**Smoking**	115/307 (37.5)	42/133 (31.6)	73/174 (42)	**.063**
**Homeless**	6/321 (1.9)	3/135 (2.2)	3/186 (1.6)	.499
**MSM**	27/292 (9.2)	13/130 (10.0)	14/162 (8.6)	.691
**Comorbidity**				
• **NAFLD**	71/292 (24.3)	36/132 (27.3)	35/160 (21.9)	.285
• **Diabetes Mellitus**	24/320 (7.5)	10/137 (7.3)	14/183 (7.7)	.906
• **Hypertension**	70/322 (21.7)	40/137 (29.2)	30/185 (16.1)	**.005**
***Viral factors***				
**HBeAg status positive**	63/276 (22.8)	34/119 (28.6)	29/157 (18.5)	.**048**
**ALT level > ULN**	119/300 (39.7)	52/128 (40.6)	67/172 (39.0)	.770
**HBV DNA (IU/mL)**				
• **< 2000**	111/203 (54.7)	46/97 (47.4)	65/106 (61.3)	**.047**
• **2000**–**20,000**	29/203 (14.3)	13/97 (13.4)	16/106 (15.1)	.731
• **> 20,000**	63/203 (31.0)	38/97 (39.2)	25/106 (23.6)	**.016**
**Fibrosis (kPa)**	5.3 ± 3.0	5.4 ± 3.3	5.2 ± 2.2	.181
**Co-infections**				
• **HCV**	12/273 (4.4)	4/113 (3.5)	8/160 (5.0)	.396
• **HIV**	36/229 (15.7)	14/91 (15.4)	22/138 (15.9)	.910
• **HDV**	6/133 (4.5)	1/51 (2.0)	5/82 (6.1)	.254

Abbreviations: IDU: Intravenous Drug Use; IU: international units; MSM: Men who have Sex with Men; NAFLD: Non-Alcoholic Fatty Liver Disease; HBeAg: Hepatitis B e Antigen; ALT: alanine aminotransferase; ULN: Upper Limit of Normal; HBV: Hepatitis B Virus; HCV: Hepatitis C virus; HDV: Hepatitis D Virus.

All values are given as frequencies n (%) or median ± IQR unless stated otherwise.

Definitions.

Alcohol abuse: > 14 units/week for men and >7 units/week for women.^21^ Patients who died or achieved HBsAg seroclearance were excluded from this analysis.

**Table 4:** Stepwise forward analyses in patients with chronic/unknown hepatitis B virus infection for being lost to follow-up as outcome variable.

Factors significantly associated with loss to follow-up on univariate analysis				P		
**Age**				.002		
**Imprisonment**				.033		
**Asian ethnicity**				.016		
**Hypertension**				.005		
**Positive HBeAg**				.048		
**HBV DNA level > 20,000 IU/mL**				.016		
**Stepwise forward analysis**		**B**	**SE**	***p***	**OR**	**95% CI**
**Step 1**	**Constant**	,949	.485	.050	2.584	
	**Age**	−.030	.013	.016	.970	.946–.994
**Step 2**	**Constant**	1.302	.520	.012	3.676	
	**Age**	−.033	.013	.010	.967	.943–.992
	**HBV DNA level > 20,000 IU/mL**	−.779	.377	.039	.459	.219–.962
**Step 3**	**Constant**	1.581	.551	.004	4.861	
	**Age**	−.035	.013	**.008**	.966	.941–.991
	**HBV DNA level > 20,000 IU/mL**	−.82	.34	**.033**	.440	.207–934
	**Asian**	−.783	.400	**.050**	.457	.209–1.00

Abbreviations: HBV: Hepatitis B Virus; CI: confidence interval; IU: international Units; SE: standard error, OR: Odds Ratio.

Factors excluded in the forward stepwise analyses are: Imprisonment, hypertension smoking and positive HBeAg**.**

### 3.5 Linkage to care and loss to follow-up over time

**Fig. 3:**
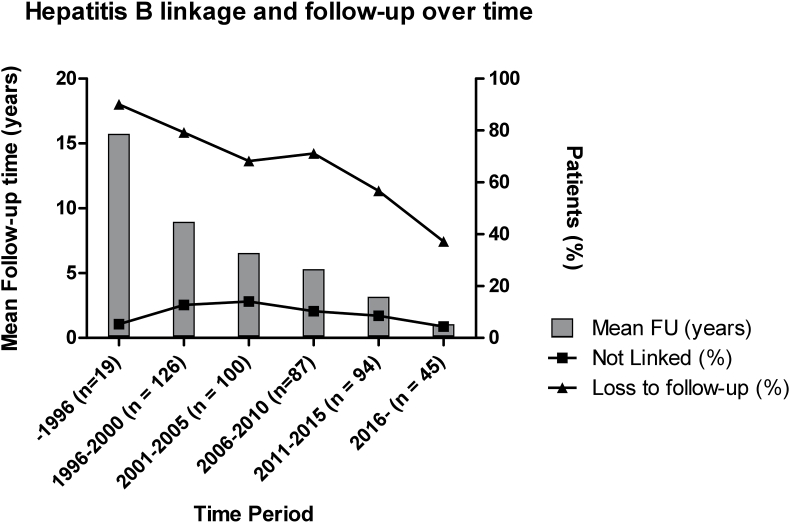
Proportion of individuals with chronic hepatitis B virus infection not linked to care or loss to follow-up over period 1996–2018 (n=471). Abbreviations: HBV, Hepatitis B virus. Definitions: not linked to care: HBsAg-positive patients without an infectious disease specialist or hepatologist evaluation; loss to follow-up, no specialist evaluation >1 year with previous evaluation.

The percentage of patients who are not being linked to care and loss to follow-up decreased over time from 12.7% in 1996 to 4.4% in 2018 and from 79.2% in 1996 to 37.2% in 2018, respectively. Fig. 3 shows the proportion of patients not being linked to care and loss to follow-up over time from 1996 to 2018.

## 4 Discussion

Assuming that interventions in viral hepatitis screening and treatment continue at the current level, 19 million hepatitis-related deaths would be anticipated between 2015 and 2030. The World Health Organization (WHO) has therefore set an action plan to eliminate viral hepatitis as a public health threat by 2030.^26^

Knowledge regarding the cascade of care is important to obtain. Understanding the gaps in linkage to care and follow-up, the needs and points of interception in optimizing HBV treatment become evident. Predictors for not being linked to care and loss to follow-up can be used to determine subgroups that need additional support for achieving optimal care. Improving the cascade of care can be achieved by point of care testing of risk groups, out of hospital screening and education of individuals at risk by specialized nurses.^27^, ^28^, ^29^, ^30^

This study determined the cascade of care in viral hepatitis B patients and identified predictors for not being linked to care and loss to follow-up in the Netherlands, a low endemic country for hepatitis B. The findings of the current study can be summarized as follows: 1) more than half of the HBsAg-positive patients did not receive the appropriate assessment of ALT, HBV DNA and HBeAg testing within 6 months after positive HBsAg test result, 2) 24% of the chronic/unknown HBV patients were not linked to care and 59% were loss to follow-up, 3) Caucasian patients were more likely not being linked to care and, 4) older age, Asian ethnicity and a HBV DNA level >20,000 IU/mL were negative predictors of loss to follow-up, 5) The percentage of patients not linked to care and loss to follow-up decreased over time, from 12.7% in 1996 to 4.4% in 2018 and from 79.2% in 1996 to 37.2% in 2018, respectively.

Our finding of suboptimal laboratory assessment was in line with previous studies that found ALT testing in 97–98%, HBV DNA testing in 44–56% and HBeAg testing in less than 52% of HBsAg-positive patients within six months after diagnosis.^31^^,^^32^ In our study, 77% had ALT testing, 42% had testing for HBV DNA and 80% had HBeAg testing. The accurate use of laboratory diagnostics can help differentiate those with need of antiviral therapy from those who need to be monitored for disease progression. Suboptimal laboratory testing as found in our study, can be a result of patient-related factors as well as healthcare worker-related factors.^17^^,^^33^

In line with previous findings, we found that an important part of the study population was not being linked to care and was loss to follow-up.^31^^,^^34^, ^35^, ^36^, ^37^ More importantly, only 25% of the total population achieved HBV DNA suppression. Confirming the suboptimal cascade of care in HBV patients, a unique point from the current study was that we also identified those patient-related factors associated with not being linked to care and with a higher risk of loss to follow-up.

To our knowledge, no studies in Europe have described predictors of not being linked to care and predictors of loss to follow-up to this extent. Studies conducted in America and Japan showed a higher linkage to care among non-Caucasian patients.^36^^,^^38^ They did not describe a lower linkage among the Caucasian population, but the percentage of Caucasians in their population was very low. Compared to the Asian population, in our Caucasian study population there was more illicit drug use, IDU and homelessness, which could in part be an explanation for lower linkage to care amongst Caucasians in our study. This may direct health care workers attention to these vulnerable cohorts. However, these factors were not identified as independent risk factors for not being linked to care in our multivariate analyses. Our finding of Caucasian ethnicity as a predictor for not being linked to care and Asian ethnicity as a negative predictor for being lost to follow-up might be explained by two hypotheses: (1) in low-endemic regions such as Western Europe, only a small part of the general population has been in contact with HBV infection and subsequently knowledge about HBV infection is limited and (2) the attitude towards hepatitis B and healthcare in general differs among different ethnicities.^39^^,^^40^

Our data also showed that older age at time of diagnosis, Asian ethnicity and an HBV DNA level >20,000 were negative predictors for loss to follow-up, meaning that those patients were less likely to be loss to follow-up. Subsequently, carefulness is advised when treating younger patients and those with a lower HBV DNA. Although a low HBV DNA is associated with the carrier state, rather than active disease, one should beware of the risk for reactivation and the importance of regular monitoring.^9^ In the study of Tang et al., a higher retention in care was also seen in Asians, compared to non-Asians.^36^ While not being assessed as predictor for loss to follow-up, older age and a HBV DNA level >2000 IU/mL have been described as positive predictors regarding the treatment of hepatitis B.^41^ In line with our findings, Assemie and colleagues found that HIV-patients aged over 45 years had a significantly lower chance of being loss to follow-up compared to those aged 15–28.^41^, ^42^, ^43^ This finding is also supported by other studies on HIV.^44^^,^^45^

In our study, not being linked to care and loss to follow-up, decreased over the years 1996–2018. In 1996, 12.7% and 79.2% of patients were not linked to care and loss to follow-up respectively. This improved, with 4.4% of patients not being linked and 37.2% loss to follow-up 2018, respectively. These data suggest that, although a substantial number of patients is not linked or retained into care, numbers are improving over time. A possible explanation for this improvement, could be better training programs in health care workers, such as specialized nurse practioners, electronic patient files and better treatment options.^46^ This improvement has also been suggested in large HIV cohorts, but a study conducted in the United States showed no significant improvement in linkage to care or treatment initiation.^47^^,^^48^

To optimize the cascade of care in chronic HBV patients, interventions are required along the whole cascade. Our HBV infected study population with a high proportion of immigrants, (ever) imprisoned patients, IDU and MSM was in line with other studies conducted in HBV population.^11^^,^^49^ Better knowledge of hepatitis B can improve linkage to care. Improved knowledge is important in both patients and healthcare workers. General practitioners can play a crucial part in this process, since being the first in line when treating a patient.^50^, ^51^, ^52^, ^53^ In our analyses of healthcare worker-related factors for patients not being linked to care, we found that general practitioners make up a large part of the requested HBsAg tests with a positive result but without further linkage to care. Better awareness amongst general practitioners in whom to test and what to do with a positive result could lead to better linkage to care.^54^, ^55^, ^56^

## 5 Limitations

This study had some limitations. First, inherent to retrospective design of the study, not all data were available. Missing data were mostly found in the group of patients not linked to care, which might cause information bias in the analyses conducted between patients linked and not linked to care. However, data collection was performed in depth in all patients, so the effect of information bias is thought to be limited. Second, it is unclear whether patients who were loss to follow-up in our hospital, are being monitored outside our centre. While the MUMC is the only hospital in Maastricht region, it is possible that some patients have moved outside this region. Third, this study focused on hepatitis B patients in the Maastricht region, so it is unclear whether the results are applicable for the rest of the Netherlands. However, the demographics of the population in Maastricht are similar to the Netherlands concerning male sex (47.9% vs. 49.6%), mean age (42.8 vs 41.8 years) and ethnicity (Caucasian ethnicity 88.9% vs. 86.9%, Asian ethnicity 7.3% vs. 5.3%, African ethnicity 3.2% vs. 3.9%) so the influence of demographic factors is thought to be limited.^57^ There are however higher endemic areas within the Netherlands, such as Amsterdam and Rotterdam, for which these results might be less applicable. This study did not focus on socio-economic status, which might also be of influence on linkage to care and loss to follow-up in this cohort.

## 6 Conclusion

In conclusion, these data suggest that to a considerable amount of HBsAg-positive individuals in the Netherlands, the recommended laboratory testing and/or specialist evaluation was not effectuated properly. Although numbers of linkage to care and follow-up improved over the last decades, our study showed that a substantial group of HBsAg-positive individuals were not linked to care or were loss to follow-up. These results reinforce the need of continual public health efforts to re-evaluate chronic HBV patients and to ensure adequate follow-up. This study has shown that ethnicity plays a role in linkage to care and loss to follow-up. Further research, national multicentre prospective in design, is needed to confirm the results of our study. The CELINE-project, a large scale retrieval project regarding Hepatitis C in the Netherlands is rolled out at this moment.^58^ We intend to perform a second study in which patients whom were identified as ‘not-linked’ or ‘LTFU’ in the study described above, will be retrieved back into care at our hospital. A similar national based project is needed in order to eliminate HBV by 2030.^59^

## Conflicts of interest

ÖK received travel grants from Gilead Sciences and his institution received grants from Gilead Sciences, AbbVie, MSD and CyTuVax B.V. AO has received honorarium for lectures from GSK and Janssen-Cilag, all payments were invoiced by the department of Medical Microbiology, Maastricht UMC+. All were outside the submitted work. The following authors reported no conflicts of interest: EO, IL, RA, DP and GK.

## Funding

This research did not receive any specific grant from funding agencies in the public, commercial, or not-for-profit sectors.

## Declaration of competing interest

The authors declare that they have no known competing financial interests or personal relationships that could have appeared to influence the work reported in this paper.
